# Distinct Roles of Nanoparticle Incorporation and Polymer Degradation in Fibroblast Response to Device Design

**DOI:** 10.1007/s44174-026-00689-y

**Published:** 2026-03-24

**Authors:** Kendell M. Pawelec, Erik M. Shapiro

**Affiliations:** 1 Department of Radiology, Michigan State University, East Lansing, MI 48824, USA; 2 Institute for Quantitative Health Sciences and Engineering, Michigan State University, East Lansing, MI 48824, USA; 3 Department of Biomedical Engineering, Michigan State University, East Lansing, MI 48824, USA; 4 Department of Chemical Engineering and Materials Science, Michigan State University, East Lansing, MI 48824, USA; 5 Department of Physiology, Michigan State University, East Lansing, MI 48824, USA

**Keywords:** Fibrosis, Nanoparticles, Degradation, Foreign body reaction, Polymer devices

## Abstract

Fibrotic encapsulation of implanted biomedical devices represents the highest cause of device failure across numerous fields, including orthopedics, breast implants and neural electrodes. While most research has focused on the immune system for driving the foreign body response and ultimate device success, fibroblasts are emerging as another key regulator of tissue response. Therefore, in the present study, we evaluated the activation of primary human dermal fibroblasts into myofibroblasts, a contractile cell that is a hallmark of fibrosis, in response to the design of porous scaffolds mimicking regenerative medicine implants. Over four weeks in culture, two factors were studied (1) nanoparticle incorporation into scaffolds for imaging functionality and (2) the scaffold’s polymer matrix, which dictates chemistry, mechanics and degradation. Incorporation of tantalum oxide (TaO_x_) nanoparticles, a radiopaque nanoparticle able to impart CT-visibility to radiolucent polymers, had a minimal effect on fibroblasts at ≤ 20 wt%, but significantly down-regulated expression of alpha smooth muscle actin (*α*SMA) (0.31 ± 0.1 fold change) compared to scaffolds without nanoparticles, likely driven by the increase in nanoscale surface roughness. Degradation rate of the polymer matrix altered myofibroblast activation, with a fast-degrading polymer, poly(lactide co-glycolide) (PLGA) 50:50, significantly up-regulating multiple myofibroblast markers, including *α*SMA (5.16 ± 2.5), vinculin, integrin *β*_1_, and integrin *β*_5_, compared to non-degrading polycaprolactone (PCL). The effect was due to the release of degradation products, namely lactic acid, which affects cellular metabolism. Together this highlights that scaffold design affects biological response immediately post-implantation in ways that impact the ultimate success or failure of biomedical devices.

## Graphical Abstract

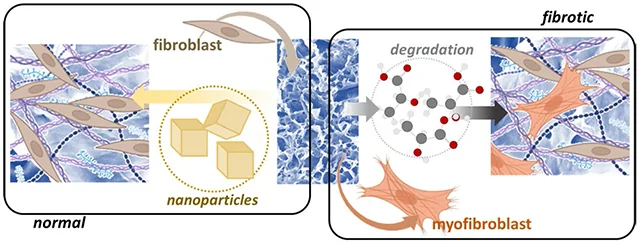

## Introduction

The biological response to implanted biomedical devices, known as the foreign body response, is a highly orchestrated interplay between device and cells, involving proteins and cytokines [1]. This complex process begins immediately post-implantation as proteins absorb onto device surfaces, subsequently triggering an immune response [1, 2]. What follows are a series of steps including the deposition of extra-cellular matrix (ECM) and the activation of fibroblasts and other mesenchymal cells into myofibroblasts, that exert contractile forces on the ECM to close wounds [3]. With the eventual maturation and remodeling of surrounding tissues to achieve normal function, the foreign body response is analogous to normal wound healing [4]. However, when acute inflammation and myofibroblast activity persist as a chronic response, biomedical devices can become encapsulated, or coated in a thick layer of ECM, that is a hallmark of pathological wound healing and fibrosis [2].

The triggers of fibrotic encapsulation around implanted devices are not well understood. However, encapsulation exerts a devastating impact on performance that can ultimately lead to failure across a spectrum of devices [2]. In orthopedics and breast implant surgery, encapsulation of devices contributes to movement of the device, pain and ultimately failure [5, 6]. Where devices function as intra-neural electrodes or sensors, a fibrotic tissue layer disrupts electrical signals, and therefore patient outcomes [7]. In all cases, failure due to fibrotic encapsulation is prevalent, and requires revision surgeries that increase the economic and societal burden of healthcare.

Given the high incidence of peri-implant fibrosis, it is important to understand which factors drive encapsulation around devices. In many cases, studies have focused on inflammatory processes as the key driver of fibrosis, since neutrophils, monocytes and macrophages are the first cell types to react to foreign materials [1]. Increasing evidence has shown a role for fibroblasts in the foreign body reaction, as they have an ability to exchange information with the immune system and modulate local and systemic responses [4]. Fibroblasts are also a major contributor to the myofibroblast population that is a hallmark of fibrotic diseases [3].

The materials properties of biomedical devices, including stiffness and surface topography, are known to affect both the immune response and severity of fibrosis [2]. Given their role in peri-implant fibrosis, it is important to understand how fibroblasts react to material properties within tissue engineering devices. This class of biomedical devices are porous scaffolds, often made from polymers, with properties that are tuned via matrix chemistry or architectural features. We hypothesized that the effects of matrix chemistry would play a significant role in driving a transition to a myofibroblast phenotype, focusing on (1) nanoparticle additions to a polymer matrix and (2) the degradation rate of the matrix.

Nanoparticle additions to polymer matrices have been employed for a variety of purposes, such as carriers for therapeutic delivery [8], as antimicrobial agents [9], or as contrast agents for medical imaging [10]. In all cases, nanoparticles must be biocompatible, preferably eliciting a minimal inflammatory response when used in implanted devices, and have the ability to be homogeneously incorporated within the polymer. Therefore, a biocompatible tantalum oxide (TaO_x_) nanoparticle was chosen. It has been demonstrated to confer radiopacity when incorporated into radiolucent polymers [11, 12], and at up to 20wt% tantalum incorporation has shown only minor inflammatory responses from macrophages [13]. While functionality of the nanoparticles after incorporation is important, this study examined the effects of nanoparticle addition within a non-degrading polymer matrix, stressing alterations of the matrix properties as the driver for biological response.

To study the effects of matrix properties on peri-implant fibrosis, porous polymer matrices mimicking biomedical devices were cultured in the presence of human dermal fibroblasts. This allowed for an in-depth investigation into the effect of each variable on fibroblast differentiation. Activation of myofibroblasts was quantified after the incorporation of 0–20wt% TaO_x_ nanoparticles into a non-degrading polycaprolactone (PCL) matrix, and as a function of the polymer matrix. While a polyester matrix was used in all cases, the polymers chosen exhibit a range of mechanical properties and degradation profiles ranging from nondegrading PCL, slow degrading poly(lactide co-glycolide) (PLGA) 85:15, to fast degrading PLGA 50:50. It was found that TaO_x_ nanoparticle addition decreased myofibroblast activation, while polymer degradation products, particularly lactic acid, could promote increased activation via metabolic modulation. Together this offers insight into the complexity of the foreign body response and considerations when designing the next generation of biomedical devices.

## Experimental

### Materials

Unless noted, all materials were purchased from Fisher Scientific. The polymer matrices used were polycaprolactone (PCL), poly(lactide-co-glycolide) (PLGA) 50:50 and PLGA 85:15. PCL (Sigma Aldrich) had a molecular weight average of 80 kDa. PLGA 50:50 (Lactel/Evonik B6010-4) and PLGA 85:15 (Expansorb^®^ DLG 85-7E, Merck) were both ester terminated and had a weight average molecular weight of 80–90 kDa. All three are biocompatible polymers used in clinically approved implants.

### Tantalum Oxide (TaO_x_) Nanoparticles

Hydrophobic TaO_x_ nanoparticles (3–9 nm diameter) were manufactured based on our previous procedure [11]. Briefly, a solution of IGEPAL^®^-CO-520 [poly(oxyethylene)nonyl-phenyl ether), Mn 441], cyclohexane and ethanol was made and a 100 mM solution of sodium hydroxide added to form a micro-emulsion, after sonication in a water bath. Next, tantalum (V) ethoxide, (Ta_2_O_5_) was introduced and stirred at ambient temperature for 20 min. At this stage, 2-[Methoxy (polyethyleneoxy)-9–12-propyl] trimethoxysilane (PEG-Silane, tech-90, MW 591–723, GELEST INC.) followed by hexadecyltriethoxysilane (HDTES, Gelest Inc., cat no SIH5922.0), an aliphatic organosilane, were added to the reaction in rapid succession. The suspension was stirred at room temperature for 16 h, then diluted with 1:1 mixture of Ethyl Ether (Anhydrous, Certified ACS, Fisher Scientific) and hexane (meets ACS specifications, VWR Chemicals). Once the reaction was complete, the nanoparticles were isolated by centrifugation as an oily pellet and purified by exhaustive dialysis in water using 12–14 kDa MWCO dialysis bags, followed by lyophilization to yield nanoparticles as a dry powder.

This process has been shown to produce hydrophobic TaO_x_ nanoparticles that can be incorporated into polyester composites with homogeneous distributions [12]. No cytotoxicity is observed in mouse tissue when exposed to low doses of hydrophobic TaO_x_ with HDTES coating, up to 20 weeks in vivo [13, 14]. Further, injection of concentrated boluses of TaO_x_ nanoparticles exhibit no toxicity up to 100 mM when injected intramuscularly in mice [11], and when used as blood pool contrast agents, TaO_x_ nanoparticles have been shown to be well tolerated and non-toxic in preclinical studies at up to three times clinical dose [15].

### Composite Manufacture: Scaffolds and Films

Porous scaffolds emulating tissue engineering implants were used for 3D cell culture and polymer films, mimicking the scaffold walls, were used to study the effects of degradation products, as described previously [13], and are shown in Fig. 1a-b. The two factors chosen for study—nanoparticle incorporation and polymer matrix—were studied separately to better appreciate the magnitude of their effect on fibroblast differentiation without introducing synergistic effects of the variables. Therefore, nanoparticles were only incorporated into a non-degrading polymer matrix (PCL).

For scaffolds, polymers were solubilized in suspensions containing TaO_x_ nanoparticles (where applicable) in dichloromethane (DCM, Sigma); PCL was used at 8 wt %, while PLGA was used at 12 wt%. The amount of TaO_x_ nanoparticles (0–20 wt%) was calculated based on the weight percent of the total dry mass (polymer + nanoparticle). To introduce microporosity to the scaffold walls, sucrose (mean particle size 31 ± 30 μm, Meijer) was added to the solution, so that the final slurry was 70 vol % sucrose and 30 vol % polymer + nanoparticles. This was followed by addition of NaCl (Jade Scientific) at 60 vol% of the total polymer + nanoparticle volume to create macroporosity. The suspension was vortexed for 10 min and pressed into a silicon mold that was 4.7 mm diameter, 2 mm high. After air drying, scaffolds were removed and washed for 2 h in distilled water, changing the water every 30 min to remove sucrose and NaCl. After air drying scaffolds overnight, they were stored in a desiccator prior to use. When nanoparticles were present, incorporation was confirmed via thermogravimetric analysis (Supplemental).

Porous films were formed from PCL and PLGA 50:50. Prior to casting films, polymers were solubilized in dichloromethane (DCM, Sigma); PCL was used at 4wt %, while PLGA solutions were at 10 wt%. Again, sucrose was incorporated into the solution, so that the final slurry was 70 vol % sucrose and 30 vol % polymer. Suspensions were vortexed, then cast and dried on glass sheets. Dried films were removed and submerged in Milli-Q water to remove the sucrose, then dried for storage. In addition, non-porous PCL films were created with the same method but without the addition of nanoparticles or sucrose.

### Cell Studies

Primary human adult dermal fibroblasts were purchased from ATCC (cat no PCS-201–012), and used before passage 5 in all experiments. Fibroblasts were cultured in complete media (Dulbecco's modified Eagle's medium (DMEM, ThermoFisher Scientific, 11–965-092), 10% fetal bovine serum (FBS, Life Technologies, A5669501), and 1% Penicillin– Streptomycin (cat no 1510122)). As a positive control for myofibroblast differentiation, cells were cultured in differentiation media: complete media supplemented with 10 ng/ml tissue growth factor beta one (TGF*β*1, Peprotech, cat 100–21-10UG).

#### Scaffold culture:

Dry scaffolds were sterilized individually, by immersing the scaffold in 70% ethanol for 60 min. In addition, PCL scaffolds were centrifuged for 5 min at 11,600xg at the start of incubation. Following ethanol soaking, scaffolds were washed in sterile phosphate buffered saline (PBS) and centrifuged for 10 min at 11,600xg. The scaffolds were stored in fresh sterile PBS until use. An hour prior to use, scaffolds were immersed in complete media. The groups tested are shown in Fig. 1c.

For cell seeding, sterilized scaffolds were placed in dry individual wells of a 48 well plate (non-treated). Fibroblasts (1 × 10^5^ cells) were pipetted onto the scaffolds in 5 μl media. A highly concentrated cell suspension was chosen to ensure that the majority of cells penetrated into the scaffold, which was already hydrated with complete media. The cells were placed in an incubator at 37 °C, 5% CO_2_ for 4 h. At that time, 400 μl of complete media was added to the well, submerging the scaffold and cells. For the positive control, cells received 400 μl of differentiation media instead. Media was changed 3 times per week for the duration of the study. Samples were harvested at day 1, day 14 and day 28 for mechanical testing and cell number quantification. Protein expression was evaluated after 14 and 28 days.

#### Film culture:

The films used for culture were composed of either PCL or PLGA 50:50 with 0 wt% TaO_x_, as shown in Fig. 1d. Dry films were cut into 13 mm diameter disks and were sterilized by immersing in 70% ethanol for 30 min, followed by two washes with sterile water. For in vitro cell tests, films were placed into 24-well inserts (CellCrown^™^, Z681903-12 EA). To reduce the leakage of media around the insert, each sample consisted of two layers: a bottom layer of non-porous PCL (cut to a circle of 24 mm diameter) and a top layer of porous film (diameter: 13 mm). After assembly into the inserts, they were placed at the bottom of a 24 well tissue culture plate, and the entire assembly was sterilized for 30 min under UV light. Sterile inserts were stored at room temperature in a biological cabinet, in sterile water, until use.

Prior to the start of culture, sterile inserts were incubated with 200 μl of complete media for one hour. After removing the media, each well was seeded with 5 × 10^4^ fibroblast cells in 400 μl complete media. Cells were placed in an incubator (37 °C, 5% CO_2_) for 4 h to allow attachment. Then the media was changed to 400 μl complete media with 0, 0.01, 0.1, 1 or 10 mM lactic acid (Sigma Aldrich, cat no 252476). As a positive control, cells were treated with differentiation media. For all tests with lactic acid and the positive control, cells were cultured on PCL films; an additional group was cultured on PLGA 50:50 films with 0 mM lactic acid. After two weeks, films were harvested for quantification of protein expression.

### Mechanical Testing

The compressive modulus of scaffolds with cells was measured at day 0, day 14 and day 28. In all cases, scaffolds without cells (incubated under the same conditions as other samples) were used as controls to assess the effects of cellular activity. Prior to performing mechanical tests, scaffold thickness and diameter was measured using a micrometer. Scaffolds were placed in a 35 mm Petri dish containing 2 ml PBS, situated within the mechanical tester (TAXT plus Texture analyzer, Stable Micro Systems, 5 kg load cell). A plastic coverslip was placed over the top of the sample before compressing to 40% of their original thickness, at 1 mm/min and then unloading. The start of the loading cycle was defined as when the force of 0.03N was reached. The loading cycle was repeated twice. The compressive modulus was calculated as the initial slope of the stress–strain curve from the first loading cycle. The percentage of deformation was obtained by dividing the scaffold thickness after the initial loading cycle by the original thickness × 100%.

After testing, scaffolds were removed from the machine, and placed in the microcentrifuge tubes on ice. After all samples were tested, they were washed in 1 ml deionized water for 5 min. The water was subsequently removed and the scaffolds were frozen at −80 °C, in preparation for cell proliferation testing. Results are presented as mean ± standard deviation.

### Cell Attachment and Proliferation

Increases in DNA over time, from cells within scaffolds, were quantified and interpreted as proliferation. DNA quantification on digested scaffolds was chosen over other cell metabolic measurements, like MTS, to minimize the effect of the 3D structure, which can hinder diffusion of dyes into and out of the scaffold and skew results. Scaffolds were harvested after 24 h, and on days 14 and 28. Samples underwent mechanical testing and then were stored at −80 °C for DNA quantification using Quant-iT^™^ PicoGreen^™^ dsDNA Assay Kit (ThermoFisher Scientific), per manufacturer's instructions. Frozen scaffolds were digested in 200 μl papain buffer (0.1 M phosphate buffer, 10 mM L-cysteine, 2 mM Ethylenediaminetetraacetic acid (EDTA), 3U/ml papain) at 60 °C overnight. In the assay, samples were diluted 1:4 in TNE buffer and loaded on a black 96 well plate. An equal amount of PicoGreen dye (1:200 in TNE buffer) was added to each well, and fluorescence was read at Ex: 480 nm, Em: 520 nm using a BioTek plate reader. Two standards were used: a cell standard (0–5 × 10^4^ cells) of fibroblast cells, created on the day of experimental cell seeding, and a DNA standard (1 μg/ml–1 mg/ml) provided by the kit. To ensure the reading was not caused by background fluorescence from the polymer inserts, scaffolds incubated for the same amount of time in complete media but without cell culture were run and data was normalized to these blanks. All tests were done in triplicate, with two technical replicates each, and presented as mean ± standard deviation.

### Protein Expression

Protein was harvested from scaffolds and films in Ripa buffer. Protein concentration was measured (BioRad, BCA kit) and expression protein markers was quantified using sodium dodecyl sulfate polyacrylamide gel electrophoresis (SDS-PAGE) and Western blotting. Full protocol details and images of complete blots are presented in Supplemental data. Briefly, samples with 2 × Laemmli buffer were incubated at 60 °C for 20 min, and separated by SDS/ PAGE. After transfer to PVDF membranes (iBlot 2 Gel Transfer Device, Invitrogen), membranes were blocked with 5% dried milk in PBST (phosphate buffered saline pH 7.4, containing 0.1% Tween-20) and probed with primary antibodies specific for alpha smooth muscle actin (*α*SMA, 1:1000, Abcam, ab7817), collagen Type I (1:1000, Life Technologies, PA595137), integrin *α*_V_ (1:1000, Abcam, ab302640), integrin *β*1 (1:1500, Abcam, ab179471), integrin *β*5 (1:500, Invitrogen, PA5-50,991), and vinculin (1:10,000, Abcam, ab129002) at 4 °C overnight. These proteins were identified as either myofibroblast markers, cellular adhesion machinery, or in the case of collagen Type I, a measure of extra-cellular matrix (ECM) production. The next day, the membrane was washed and incubated with anti-mouse IgG-HRP (Millipore, 12–349) or anti-rabbit IgG-HRP (ab205718, Abcam) at room temperature for 2 h. Protein bands were visualized with Amersham ECL Plus substrate (cytiva, RPN2232) on a Li-Cor Odyssey FC and resulting signal was quantified via Image J.

Developed membranes were washed in PBS and stripped for 15 min in Restore Western Blot Stripping Buffer (Thermo Scientific 21,059) before being either blocked and reprobed, or stained for total protein using the BLOT-Fast Stain (G Biosciences, cat# 786–34) according to manufacturer's directions. The total protein signal was imaged under visible light on a c300 imager (Azure biosystems). Protein expression was quantified via Image J and normalized to total protein to adjust the measured band intensity. For comparisons between membranes, all signals were normalized to PCL + 0 wt% TaO_x_, which was run on every membrane. In graphs of protein expression, a dashed line is used to indicate no difference in expression (fold change of 1) compared to the control group of PCL + 0 wt% TaO_x_ (no TGF*β*) at week 2. For easier comparison, the control group remained the same across all graphs of protein expression. In addition, graphs with all groups plotted together are presented in Supplemental data (Figure S14). All data reported are the result of three independent replicates (two technical replicates each) and are reported as mean ± standard error.

### Immunohistochemistry

Prior to staining, fixed samples were stored in PBS at 4 °C. Films were stained without further preparation. Scaffolds were embedded in Tissue Tek Optimal Cutting Temperature (OCT) Compound and frozen on dry ice. Once embedded, a Leica CM3050S cryostat was used to make 20 μm slices through the cross-section of scaffolds and adhered to glass slides. Slides were allowed to air dry, and stored at −20 °C until staining.

Prepared samples, either films or cryosectioned slices on slides, were washed in PBST, then permeabilized in 0.1% Triton X-100 in PBS, followed by two PBST washes. Samples were then blocked with 5 wt% bovine serum albumin (BSA) in PBST, for 1 h, at room temperature. After washing twice in PBST, samples were incubated with primary antibodies, overnight at 4 °C, in 3 wt% BSA/PBST. Following primary incubation and two PBST washes, secondary antibodies in PBS were incubated with the samples for 1–2 h at room temperature. To visualize the actin cytoskeleton, samples were incubated for 30 min in ActinRed^™^ 555 ReadyProbes^™^ (Invitrogen, R37112, 2 drops per ml PBS). Finally, samples were washed twice with PBS and left in a solution of PBS and DAPI stain (1:000 in PBS, ThermoFisher Scientific, 62,248). Primary antibodies, and the dilutions used, were: *α*SMA (1:300, Abcam, ab7817) and collagen Type I (1:500, Life Technologies, PA595137). Secondary antibodies: Alexa Fluor 488 (1:1000, A11070), and Alexa Fluor 647 (1:1000. A21236).

Stained samples were inverted onto a coverslip in a drop of Slow Fade Diamond Antifade Mountant (Invitrogen, S36972). Imaging was performed using a Leica DMi8 microscope, using an LASX software interface. With the high surface roughness of the samples, z-stacks were taken at a minimum of 3 places and post-processed with Thunder image analysis (Leica) to remove background fluorescence.

### Scaffold Imaging

Micro-computed tomography (μCT) imaging was performed on a Revvity Quantum GX. Scaffolds were imaged in PBS at 90 keV, 88 μA, with a 25 mm field of view at a 50 μm resolution. Samples were imaged while immersed in cell culture media, in individual wells of a 48 well plate.

Scaffold architecture was imaged via scanning electron microscopy (SEM). Cryosectioned slides were immersed in PBS at room temperature for 5 min, in 70% ethanol for 5 min and then washed in 100% ethanol and allowed to air dry to remove OCT compound. After drying, sections were transferred to 13 mm aluminum stubs with double sided carbon tape. Stubs were then sputter coated with platinum and examined using a MIRA3 Tescan, at 10 keV in scanning electron mode.

### Statistics

Statistics were performed using GraphPad 10.4.0. Data was assessed for normality with a Shapiro–Wilk test. Biological data (protein expression and proliferation) was normally distributed, and groups were compared via ANOVA, followed by Fisher's LSD test. Mechanical properties were first compared across groups using a nonparametric Kruskal Wallis test, followed by an uncorrected Dunn’s test. In all cases, *α* < 0.05 was considered significant, with a 95% confidence interval.

## Results & Discussion

In this study, regardless of the polyester, or nanoparticle addition, the porous architecture of the tissue engineering scaffolds was kept constant, as the structure of scaffolds also affects the foreign body reaction independent of material [16]. Scaffolds, seen in Fig. 2a, consisted of a macroporous architecture with mean pore size of 350–400 μm (Fig. 2b), and microporous walls (Fig. 2c) with thin struts between pores (Fig. 2d). The range of features accommodates both nutrient diffusion and cellular attachment, and fibroblasts were able to proliferate within the structure over four weeks of culture, Fig. 2e-f.

Importantly, this study confirmed that in vitro culture on porous scaffolds supported fibroblast differentiation towards a myofibroblast phenotype, a hallmark of fibrotic diseases, with the addition of 10 ng/ml TGF*β*1 in the culture media, Fig. 2g. As expected, this led to a significant increase in *α*SMA, the hallmark of myofibroblast differentiation, starting at week two and continuing throughout the culture period. In addition, by week four, supplementation led to a significant increase in collagen Type I expression, and expression of attachment integrins *α*_V_, *β*_1_and *β*_5_. Increases in collagen Type I, a key structural ECM component, signal a greater propensity for the scar formation typical of myofibroblasts [2, 17]. Indeed, it is not surprising that changes in differentiation and collagen production were linked to integrin expression changes, as integrins are known to regulate collagen genes [18].

Foreign body reactions to biomaterials are complex and often determine whether implanted devices are clinically successful or not [19]. In some of the worst cases, the initial inflammatory reaction post-implantation leads to peri-implant fibrosis, or fibrotic encapsulation, with a dense layer of ECM. When this occurs, devices cannot fulfill their primary function and must be removed, increasing patient morbidity [2]. Both macrophages and fibroblasts are essential in the foreign body response, often working in tandem [16]. The fibroblast transition to a myofibroblast phenotype presages encapsulation, although there are still many open questions surrounding the origin and sub-populations of fibroblasts that respond in foreign body reactions [1, 4].

In fibrotic tissue, TGF*β*1 is activated by integrins: *α*_V_*β*_6_ in lung and *α*_V_*β*_5_ in skin [18, 20]. It has been hypothesized that fibroblasts express the integrin *α*_V_ sub-unit in excess and control myofibroblast differentiation through expression of the *β* sub-unit [18]. In the present study, stimulation of myofibroblast differentiation correlated to an up-regulation of both the *β*_1_and *β*_5_ sub-unit, which reflects both differentiation and changes in the cellular machinery required to attach to the polyester substrate. The effect of TGF*β*1 on vinculin expression, present in focal adhesions and related to mechanical force generation in fibroblasts, was less pronounced [21]. Overall, the myofibroblast transition with TGF*β*1 is reminiscent of what is observed clinically [18].

### Cell Activity Affects Scaffold Properties

This study showed no significant differences in the initial attachment of human adult dermal fibroblasts due to polymer matrix or nanoparticle content 24 h after seeding. However, the ability for fibroblasts to proliferate within the porous structure was significantly impacted by matrix properties. The greatest proliferation was observed on matrices that were stable over time, namely those based on the non-degrading PCL [22]. In addition, over 4 weeks in culture, there was a significant increase in cell proliferation, interpreted as an increase in DNA quantity, with increasing TaO_x_ nanoparticle content, Fig. 3a. Scaffolds of PCL with 20 wt% TaO_x_ had an average of 611.1 ± 82% growth, compared to PCL without nanoparticles at 208.6 ± 109% growth. Effects of nanoparticles on both cellular attachment and proliferation with incorporation of nanoparticles have been noted in other composite systems, where they generally reach a maximum and then decline with further nanoparticle addition [23, 24]. On PLGA scaffolds, a polyester with increased degradation compared to PCL, cell proliferation occurred over the first two weeks of culture and then cell number remained constant. This is likely a result of decreasing stability of the PLGA matrix, limiting cellular attachment sites, as it begins to degrade between weeks two and four, as noted macroscopically in Fig. 3b. Stimulating the myofibroblast transition with the addition of TGF*β*1 appeared to promote greater cell proliferation over time, but the trend was not significant.

In general, increased fibroblast attachment in vitro equates to greater capsule formation around devices in vivo [25]. There are a number of cues inherent in biomedical scaffolds that can affect fibroblast attachment and differentiation, including mechanics, topography, surface chemistry and bulk material chemistry [4]. While cells could penetrate through the scaffolds, most remained near the top and side surfaces (supplemental). This was due to limitations of nutrient diffusion from cell culture media, an effect commonly observed during in vitro culture on 3D substrates. Larger scaffold pore sizes can enhance diffusion and cell infiltration, but also significantly reduce the overall surface area available for cell proliferation, requiring a delicate balance [26].

Culturing fibroblasts within the scaffolds significantly affected the scaffold properties over two weeks. This observation is in agreement with literature that highlights increased degradation of polymer scaffolds implanted in vivo compared to acellular in vitro environments, hypothesized to be due to increased fluid flows and protease concentrations [13, 27]. In the present study, macroscopic changes to scaffold dimensions were observed over four weeks of culture, Fig. 3b. This was particularly true for PLGA 50:50 scaffolds, that have the fastest degradation time of the polyesters studied, degrading within a matter of weeks [12]. While the percent change from the original diameter was mild for PLGA scaffolds (−6.10 ± 9.4%), since swelling was observed in the PLGA 50:50 control without cells, it correlated to a −31.1 ± 6.9% change in final diameter between scaffolds with and without cells, Table 1.

In addition to observing dimensional changes, matrix mechanics, including compression and deformation, were monitored over in vitro culture, Table 1. Overall, PCL scaffold mechanics were not significantly affected by cell culture over time for any condition tested. Even TGF*β*1 supplementation that drove myofibroblast transition did not affect macroscale mechanics.

On the other hand, PLGA scaffolds were significantly affected by fibroblast culture, Fig. 3b and c. Namely, scaffolds lost significant structural integrity and mechanical properties up to two weeks earlier when cells were present, compared to controls that were incubated in media without cells, Fig. 3c. PLGA is known to be sensitive to the action of proteases, which are present in complete culture media, and their expression is triggered during the foreign body response [28]. With the presence of fibroblasts, polymer chain scission was increased, observed as a significant decrease in mechanical strength of scaffolds with cells compared to acellular counterparts. In addition, swelling of the PLGA scaffolds decreased by 31% with fibroblasts present in the matrix. Swelling or contraction of PLGA scaffolds during degradation is significantly affected by pH conditions [12]. Matrix morphology can also be altered by cells exerting contractile forces on the matrix over time. This phenomenon has been most visible in scaffolds with low mechanical strength, such as collagen implants [29]. However, the role of myofibroblast activity on contraction is believed to be relatively minor, as the induction of myofibroblast differentiation on PCL was not enough to alter the macroscopic scaffold mechanics over the four weeks of culture.

By the end of four weeks, the compressive modulus of PLGA 50:50 was not significantly different than PCL, 1.81 ± 0.6 kPa and 1.90 ± 0.3 kPa, respectively. PLGA 85:15, with its slower degradation rate than PLGA 50:50, had only begun to lose structural integrity at four weeks of in vitro culture. However, the permanent deformation in PLGA scaffolds increased over time regardless of polymer blend. At four weeks, deformation reached 34.1 ± 2.9% and 44.3 ± 10% for PLGA 85:15 and PLGA 50:50, significantly greater than the initial deformation of scaffolds observed after 24 h.

### Nanoparticle Incorporation has a Limited Effect on Fibroblasts

Nanoparticles are increasingly being incorporated into polymers to provide added functionality. In the present case, TaO_x_ nanoparticles introduced into a PCL matrix conferred radiopacity to the scaffold, Fig. 4a, that scaled with nanoparticle content, as noted in literature [13, 14]. While fibroblast proliferation increased with TaO_x_ nanoparticles, they had very little effect on the protein expression of fibroblasts. At 20wt%, nanoparticles significantly reduced the expression of key myofibroblast markers over four weeks, Fig. 4b-d. In particular, the expression of *α*SMA was significantly down-regulated, reaching 0.31 ± 0.1 fold change from PCL + 0wt% TaO_x_ at week two, and suggesting a decrease in myofibroblast differentiation. Integrin *α*_V_ expression was also sensitive to nanoparticle addition, leading to significant down-regulation. No significant changes were noted in vinculin, integrin *β*_1_ or collagen Type I expression.

The change in attachment proteins on scaffolds with nanoparticles suggests that the surface of the material was altered with nanoparticle addition, and that these changes were driving the decreased differentiation of fibroblasts. In other cell types, the biological response associated with the introduction of TaO_x_ nanoparticles has been mediated by changes to the surface topography of the composite, namely increased nanoscale surface roughness [13, 24]. As in the current study, these were associated with changes in integrin expression across a variety of cell types, including glia and macrophages [13, 24]. The exposure conditions of nanoparticles to cells and tissues, as well as their size and shape, play a large role in determining the biological response, including the exposure route and time of contact [30].

In the present study, nanoparticles are not released but firmly encapsulated in a non-degrading matrix. Thus, nanoparticles are affecting biological activity indirectly via materials properties. In literature, nanoscale surface roughness has been linked to decreased fibrous capsule formation and collagen deposition compared to scaffolds with predominately microscale features [31, 32]. The reduced fibrotic deposition around implanted scaffolds has been linked, not only to increased scaffold survival, but to increased density of blood vessels [2, 32].

It is not clear if the decreased expression of myofibroblast markers is linked with altered cell attachment mechanisms. In studies on silicone implants, blocking integrin *α*_V_ reduced fibroblast activation by reducing sensitivity to substrate mechanics [33]. However, the effects of surface topography may also be rooted in changes in wettability, as more hydrophilic surfaces drive the myofibroblast transition [34]. In all cases, altered foreign body responses are likely mediated by changes in protein adsorption with surface topography, with lower adsorption leading to a limited response [4].

### Degradation Products Drive Cellular Behavior

Polyester matrices are very versatile, representing a wide range of mechanical properties and degradation profiles, but also a wide range of foreign body responses. The present study confirmed that fibroblasts attached and proliferated on all matrices tested, Fig. 5a. While nanoparticle addition had a limited effect myofibroblast markers, properties of the polymer matrix stimulated greater changes in protein expression. Of note, *α*SMA expression was significantly increased over four weeks on PLGA 50:50 scaffolds, reaching a fold change of 5.16 ± 2.5 compared to PCL. Cells on PLGA 85:15 showed a 2.5 ± 1.5-fold up-regulation, Fig. 5b-d. In addition, PLGA 50:50 stimulated higher expression of integrin *β*_1_ and integrin *β*_5_. However, at no point was marker expression of *α*SMA, or integrin *β*_5_ on PLGA 50:50 higher than the positive myofibroblast differentiation control (PCL + TGF*β*). These results track with literature where PCL and PLGA matrices implanted in vivo have been shown to trigger different immune responses [27].

Fibroblasts are able to respond to mechanical cues within their environment and can respond to changes over several orders of magnitude [33]. The range of apparent compressive modulus of the scaffolds used in the present study were between 2 and 7 kPa, Table 1. Even in this range, in line with the modulus of natural tissue, greater mechanical properties increase *α*SMA expression and vinculin organization [35]. It should be noted that the apparent modulus reflects a combination of the inherent compressive modulus of the polymer and characteristics of the scaffold architecture. Thus, fibroblasts cultured within the scaffolds may be perceiving much greater mechanical forces than recorded during testing.

However, mechanical properties of the matrix cannot explain the observed changes in protein expression. Both PLGA 85:15 and PLGA 50:50 began the study with comparable compressive moduli, and yet *α*SMA expression on PLGA 50:50 scaffolds only significantly increased after mechanical strength was lost. As noted in Table 1, by four weeks, the mechanical strength of PLGA 50:50 had fallen to the same level as PCL, and yet marker expression was significantly greater. It is more likely that the release of degradation products, such as lactic acid, drive protein expression, as mass loss begins after mechanical modulus decreases [36]. Of the polyesters studied, PLGA 50:50 has the fastest degradation rate, followed by PLGA 85:15 [12]. It is noted that marker expression on PLGA 85:15 appeared to be following the same trends as PLGA 50:50, namely promoting myofibroblast differentiation. However, cells on PLGA 85:15 had lower expression of markers, consistent with a lower level of degradation products [37]. In comparison, cell growth was similar between the two PLGAs studied, suggesting that cells were preferentially differentiating rather than proliferating.

Degradation products, and lactic acid in particular, are believed to mediate the biological response to polymers in many systems, including glia and macrophages [37, 38]. Lactic acid is part of the glycolytic pathway and can shift cellular metabolism in ways that up-regulate pro-inflammatory factors [38]. Glycolysis metabolism may also lead to an up-regulation of reactive oxygen species that induce TGF*β*1 gene expression in fibroblasts, ultimately inducing fibrosis [39]. To further test the hypothesis that lactic acid was responsible for the protein expression changes, fibroblasts were cultured on PCL microporous films, the substrate with the lowest potential for inducing myofibroblast differentiation, with lactic acid supplementation in the media. The films were designed to mimic the microporous scaffold walls, without creating gradients of oxygen or metabolites induced within 3D scaffolds, to ensure all cells experienced elevated lactic acid levels.

With the addition of lactic acid to the culture media, the expression of fibroblast markers changed significantly, Fig. 6a-b, but cells remained well spread and attached to the PCL substrates (Fig. 6c). Moderate doses of 0.1–1 mM lactic acid had the greatest positive effect on the expression of all proteins at week 2. At 10 mM, the expression of myofibroblast markers was reduced back to baseline levels, Fig. 6a-b. Films of PLGA 50:50 have been shown to have a burst release of lactic acid and di-lactic acid over the first week, reaching a cumulative release of 0.015 mM in the absence of proteases [37]. It is likely that elevated protease activity during in vitro cell culture increases the release rate of lactic acid, potentially bringing the concentration of lactic acid within the range that stimulated the greatest myofibroblast activation.

Once again, PLGA 50:50 films had increased *α*SMA expression compared to PCL films with no lactic acid supplementation. However, changes in attachment proteins on the films did not follow trends seen in three-dimensional scaffolds. The transition from 2 to 3D is known to affect cell attachment mechanisms, making it likely that differences in expression were due to additional architectural cues or local pH conditions within the confines of the scaffold that were not present during film culture [40].

Lactic acid has previously been shown to drive the myofibroblast transition in lung fibroblasts and in adipocytes, driven by changes in pH [41, 42]. In these studies, 10 mM lactic acid supplementation gave the same marker expression as TGF*β*1 after three days of culture [41]. It is hypothesized that this effect might be transitory and have peaked by the two week time point used in the present study, which was required to get comparable degradation of PLGA 50:50. The use of a microporous film substrate, compared to a traditional tissue culture polystyrene or glass may also affect protein expression of cells in culture, by altering cell shape and attachment machinery.

In addition to in vitro effects, lactic acid has been associated with pathological conditions related to fibrosis [41]. In clinical patients, elevated levels of lactate have been found in lung tissue of patients with idiopathic pulmonary fibrosis (IPF) and in the blister fluid of scleroderma patients [39, 43]. This suggests that the preclinical and in vitro results regarding the effects of lactic acid can be translated to humans. Indeed, PLGA 50:50 is known to stimulate a robust inflammatory response after implantation, although no long-term complications have been reported for clinical devices [28, 44]

Ideally, biomedical implants can be engineered to encourage the attachment of fibroblasts to anchor implants, while avoiding the processes that lead to encapsulation [34]. The ability for degradation products to stimulate biological responses is well recognized [45]. However, it is important to realize that material degradation products can affect the foreign body reaction even at the initial moments of implantation. A limitation of this study is its focus on in vitro responses. Within the in vivo environment, micro-mechanical forces play a role in determining the severity of response to implanted devices across species [17], and it is becoming increasingly apparent that the biological milieu around the device can also affect the type and extent of the foreign body reaction [14, 46]. Despite the simplified system, this study serves as a starting point for discussion around the contribution of material factors within the foreign body reaction.

With peri-implant fibrosis playing a large role in device failure, strategies have been proposed to mitigate this, including the incorporation of anti-inflammatory therapeutics into polymer matrices, such as capsaicin [2, 47]. Since myofibroblast differentiation requires lactate transporter activity and lactate dehydrogenase, inhibition of these proteins might also be good targets for a therapeutic strategy and are being tested for treating fibrotic disease [41, 48]. Whether tracking devices via in situ imaging, or delivering therapeutics to ensure tissue integration, nanoparticle incorporation into polymer matrices is poised to play a key role in the next generation of medical implants. From the current study, the use of nanoparticles may also have previously unappreciated effects and lead to a reduction in periimplant fibrosis. Together these highlight the interplay of different device characteristics in determining the foreign body response.

## Conclusions

Biomedical devices interact with native tissues via the foreign body response. While most literature has focused on the immune component of this process, this study emphasizes the role of fibroblasts in responding to biomaterials cues, such as polymer chemistry and surface topography. When human dermal fibroblasts were cultured on porous polymer scaffolds, the presence of cells increased the degradation rate of polymer scaffolds. The introduction of up to 20 wt% TaO_x_ nanoparticles into the polymer matrix, rendered the scaffold radiopaque, and led to a decrease in myofibroblast markers. The choice of polymer matrix had the most significant effect on myofibroblast activation. The fastest degrading polymer, PLGA 50:50, released lactic acid into the culture environment that could significantly up-regulate the protein markers for myofibroblasts, namely *α*SMA, reaching a 5.16 ± 2.5-fold increase over inert PCL matrices. The lactic acid released during degradation was shown to contribute to the expression of myofibroblast markers, separate from substrate mechanics. Together the addition of nanoparticles and tailored degradation of polymers are two ways that engineers can tune biomedical scaffolds to avoid the excessive per-implant fibrosis that leads to clinical device failure.

## Supplementary Material

Supplemental info

**Supplementary Information** The online version contains supplementary material available at https://doi.org/10.1007/s44174-026-00689-y.

## Figures and Tables

**Fig. 1:**
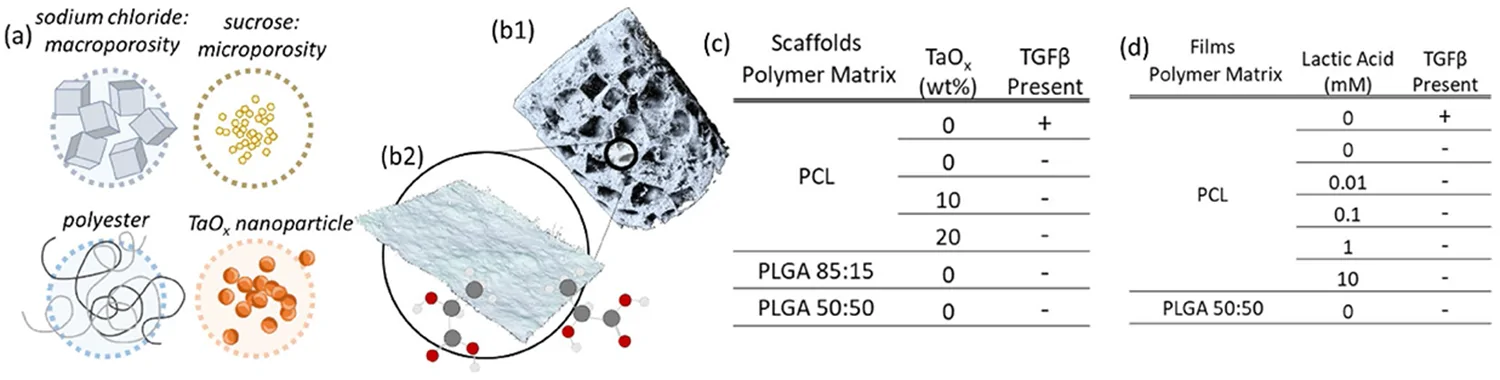
Materials in the form of both scaffolds and films were tested for biological activity. **a** Four key components were present in scaffolds, shown schematically in **b1**, and films **b2** contained only polyester and sucrose for microporosity, representing the scaffold walls. **c** Scaffolds variables studied were TaO_x_ nanoparticle incorporation and polyester matrix. **d** Fibroblasts on films of PCL were cultured with increasing lactic acid concentrations in the media to simulate conditions of degrading PLGA 50:50

**Fig. 2:**
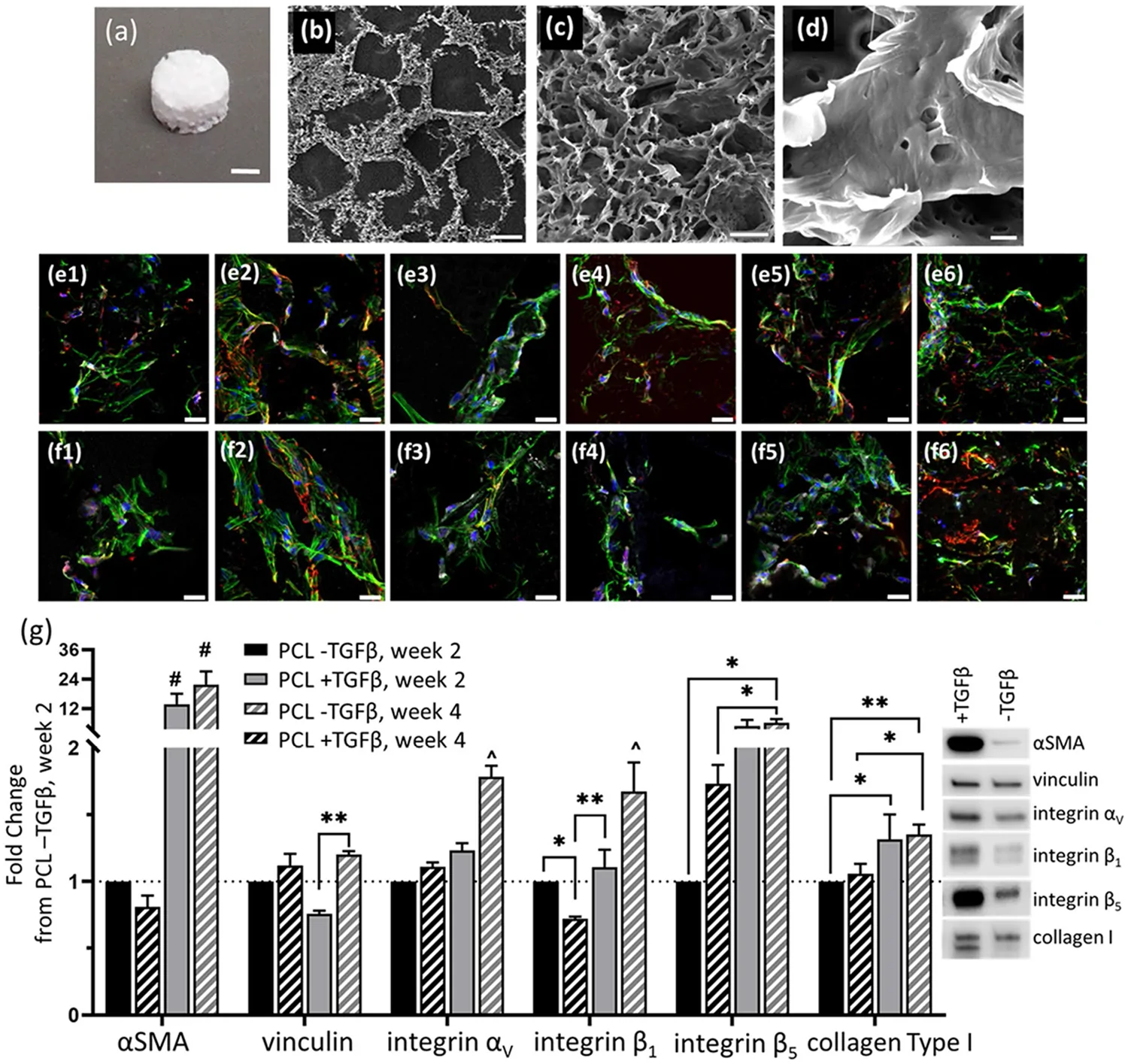
Porous tissue engineering scaffolds supported growth and differentiation of human adult dermal fibroblasts. **a** Seen macroscopically, scaffolds were designed to mimic tissue engineering implants. With electron microscopy, hierarchical features were visible, including **b** macroporosity, **c** microporosity in the scaffold walls and **d** individual struts of the micropores. The features allowed fibroblasts to grow into the scaffolds at **e** 2 weeks and **f** four weeks; red: *α*SMA, green: actin, white: collagen Type I, blue: nucleus. 1: PCL, 2: PCL + TGF*β*1, 3: PCL + 10 wt% TaO_x_ nanoparticles, 4: PCL + 20 wt% TaO_x_ nanoparticles, 5: PLGA 85:15, 6: PLGA 50:50. **g** Fibroblasts were capable of transitioning to myofibroblasts, common to fibrotic phenotypes, as shown by protein expression over four weeks, quantified by Western blotting. All expression data was normalized to PCL -TGF*β* at week 2, and protein loading on the blot was normalized by total protein loaded (supplemental). Protein expression data is presented as mean ± standard error and characteristic bands at week 4 are shown. *α* < 0.05, * *p* < 0.05, ** *p* < 0.01, # significantly different from all other groups (*p* < 0.0001), ^ significantly different from all other groups (*p* < 0.001). Scale bar **a** 1 mm, **b** 250 μm **c** 15 μm, **d** 1 μm, **e**–**f** 25 μm

**Fig. 3:**
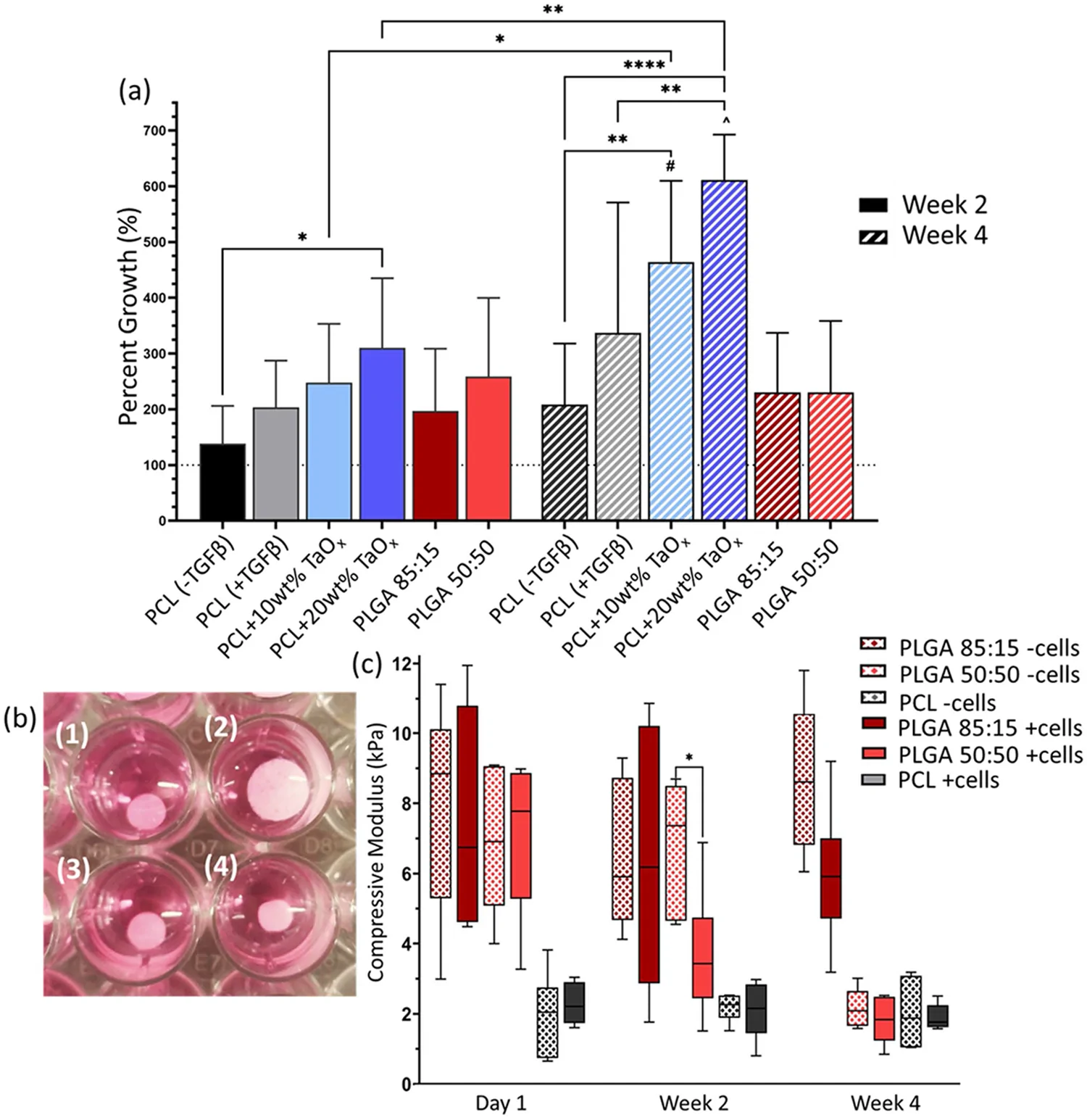
Scaffold proliferation and mechanical properties are determined by the scaffold matrix and can be affected by cellular interactions. **a** Percent growth over 4 weeks was significantly higher on composites of PCL + nanoparticles than PLGA matrices (mean ± st deviation). **b** Macroscopic effects on the scaffold dimensions were noted due to cell interactions: (1) PLGA 85:15 without fibroblasts, (2) PLGA 50:50 without fibroblasts, (3) PLGA 85:15 with fibroblasts, (4) PLGA 50:50 with fibroblasts. **c** Cell interactions led to significantly faster degradation, measured via the compressive modulus, for PLGA scaffolds; box plot whiskers show the spread of data from minimum to maximum values. *α* < 0.05, * *p* < 0.05, ** *p* < 0.01, **** *p* < 0.0001

**Fig. 4:**
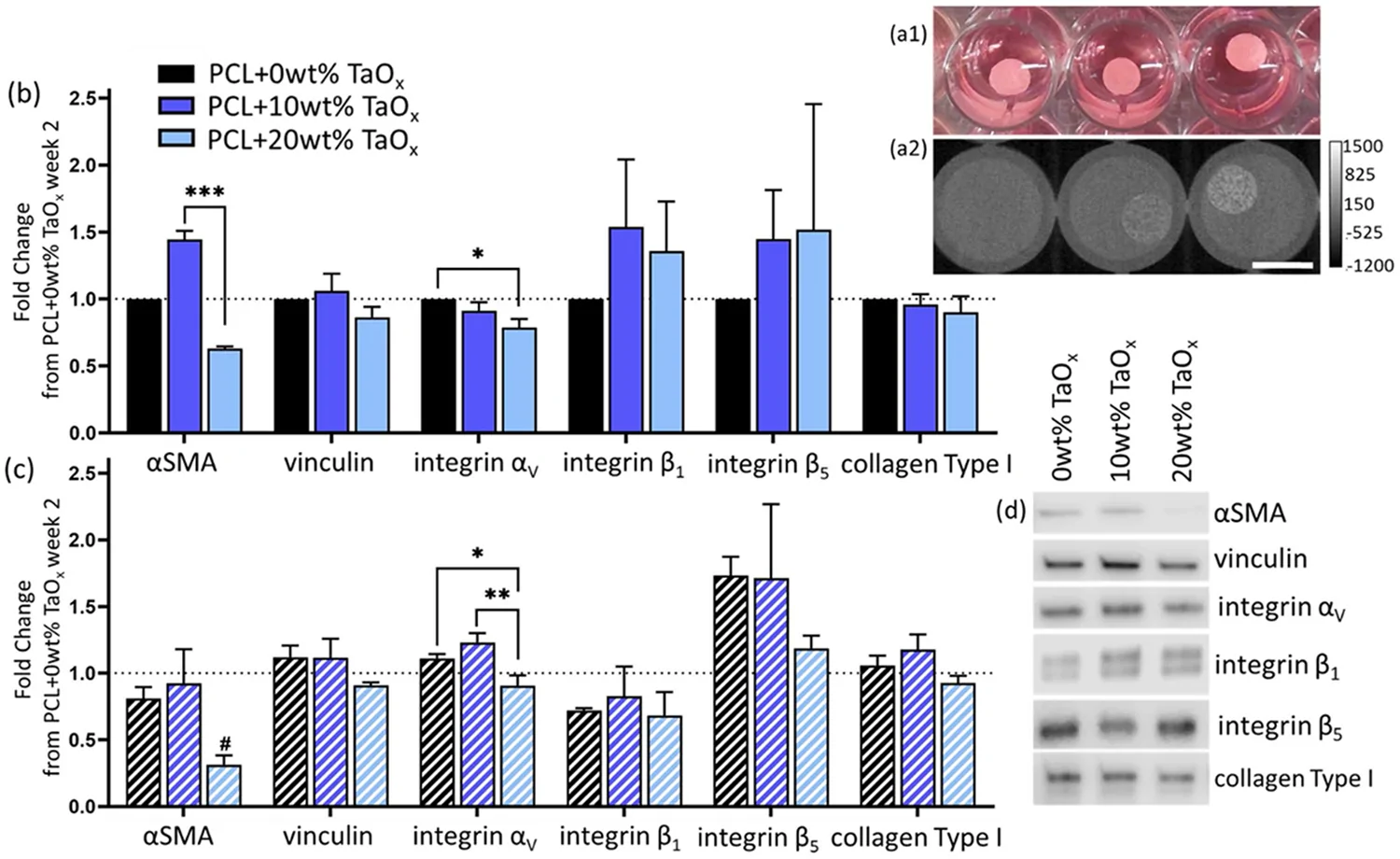
Nanoparticle addition in PCL scaffolds did not promote myofibroblast differentiation. **a** After four weeks in culture, PCL + radiopaque TaO_x_ scaffolds remained intact, seen (1) visually and (2) via μCT. TaO_x_ nanoparticle addition increased radiopacity of scaffolds; from right to left: PCL + 0 wt% TaO_x_, PCL + 10 wt% TaO_x_, PCL + 20 wt% TaO_x_. Protein expression of myofibroblast markers were either stable or down regulated with increasing TaO_x_ addition after **b** two weeks or **c** four weeks. **d** Typical Western blotting protein bands at week four for markers of myofibroblast transition: *α*SMA, vinculin, integrin *α*_V_, integrin *β*_1_, integrin *β*_5_, and collagen Type I. All expression data was normalized to PCL + 0 wt% TaO_x_ (without TGF*β*1 supplementation) at week 2, and protein loading on the membrane was normalized by total protein (supplemental). Protein expression data is presented as mean ± standard error. *α* < 0.05, * *p* < 0.05, ** *p* < 0.01, # significantly different from all other groups at this time point (*p* < 0.05). Scale bar: **a** 5 mm

**Fig. 5:**
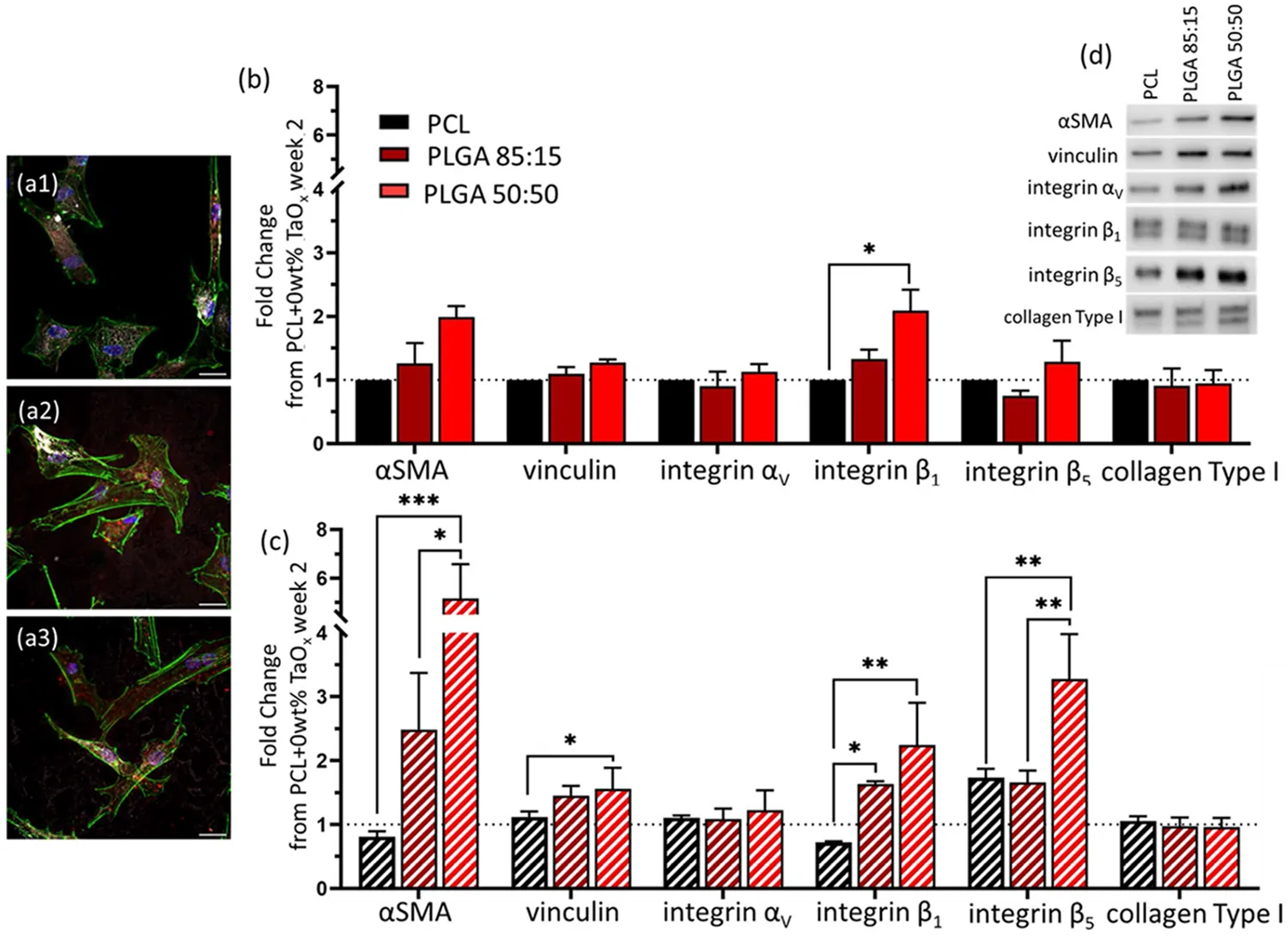
The polymer matrix making up scaffolds had a significant impact on myofibroblast marker expression over four weeks. **a** Fibroblasts were attached and well spread on the surface of all polymers after one week culture on films (1) PCL, (2) PLGA 85:15 (3) PLGA 50:50. Green: actin, red: *α*SMA, white: collagen Type I, blue: nucleus (DAPI). Protein expression was quantified via Western blotting at **b** two weeks and **c** four weeks of culture. Markers examined were *α*SMA, vinculin, integrin *α*_V_, integrin *β*_1_, integrin *β*_5_, and collagen Type I. All expression data was normalized to PCL (+ 0 wt% TaO_x_, without TGF*β*1 supplementation) at week 2, and protein loading on the membrane was normalized by total protein (supplemental). **d** Typical protein bands of markers at four weeks. Protein expression data is presented as mean ± standard error. *α* < 0.05, * *p* < 0.05, ** *p* < 0.01, *** *p* < 0.001. Scale bar: **a** 25 μm

**Fig. 6:**
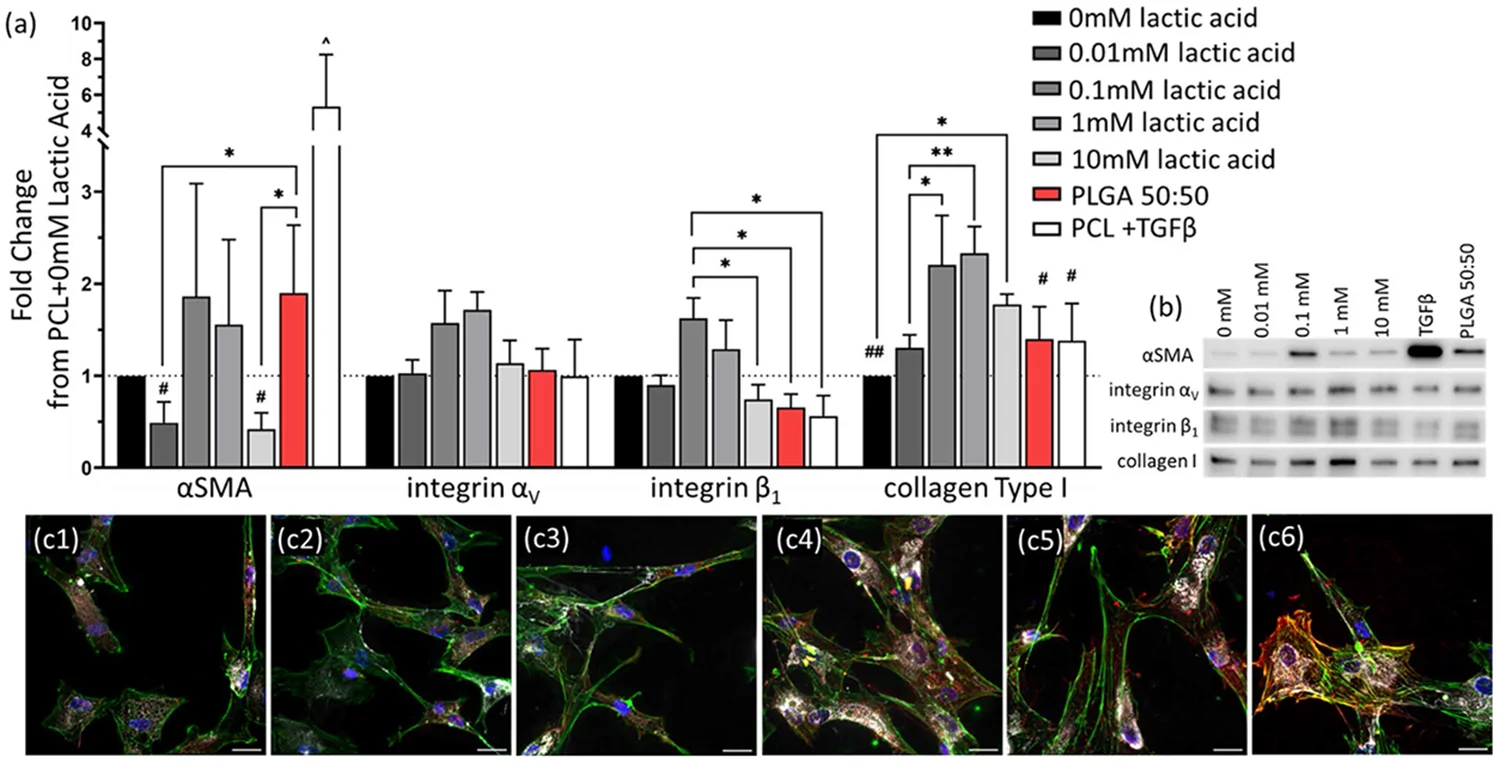
Degradation products of PLGA matrices, namely lactic acid, significantly impact fibroblasts. **a** Protein expression of fibroblasts, on microporous films after two weeks of culture, was altered by addition of 0–10 mM lactic acid, as seen in **b** typical protein bands in Western blotting. All expression data was normalized to PCL without TGF*β*1 supplementation (0 mM lactic acid), and protein loading on the membrane was normalized by total protein (supplemental). Protein expression data is presented as mean ± standard error. **c** Fibroblasts attached and were well spread regardless of media supplementation (1) 0 mM lactic acid, (2) 0.01 mM lactic acid, (3) 0.1 mM lactic acid, (4) 1 mM lactic acid, (5) 10 mM lactic acid, (6) 0 mM lactic acid + TGF*β*1 supplementation. Fibroblasts were stained for collagen Type I (white), *α*SMA (red), actin (green) and nucleus (blue). *α* < 0.05, * *p* < 0.05, ** *p* < 0.01, # significantly lower than 0.1–1 mM lactic acid (*p* < 0.05), ## significantly lower than 0.1–1 mM lactic acid (*p* < 0.01), ^ significantly greater than all other groups (*p* < 0.001). Scale bar: **c** 25 μm

**Table 1:** Mechanical properties measured over four weeks on scaffolds in contact with fibroblasts

	TaO_x_ (wt%)	TGF*β* present	Compressive modulus (kPa)			Deformation (%)		Deformation ^a^ (% change)	Final diameter^a^ (% change)	Final thickness (% change)
			Day 1	Week 2	Week 4	Day 1	Week 4			
PCL	0	+	2.27 ± 0.6	2.31 ± 0.4	2.02 ± 0.5	15.7 ± 0.8	12.6 ± 1.3	−1.39 ± 9.8	1.48 ± 2.5	6.38 ± 5.6
	0	−	2.28 ± 0.6	2.09 ± 0.8	1.90 ± 0.3	15.3 ± 1.2	12.5 ± 1.4	−2.14 ± 11	0.28 ± 1.8	3.32 ± 3.9
	10	−	2.34 ± 1.4	2.19 ± 1.1	1.44 ± 0.7	15.1 ± 1.2	14.2 ± 2.4	−0.07 ± 17	0.10 ± 1.3	2.41 ± 6.2
	20	−	1.98 ± 0.9	2.41 ± 1.0	1.92 ± 0.6	15.5 ± 1.0	12.8 ± 1.2	0.17 ± 9.7	1.82 ± 2.1	4.77 ± 1.0
PLGA 85:15	0	−	7.5 ± 2.8	6.37 ± 3.3	5.95 ± 1.8	29.5 ± 3.1	34.1 ± 2.9	4.55 ± 8.9	−1.47 ± 0.8	−4.22 ± 4.5
PLGA 50:50	0	−	7.10 ± 2.0	3.67 ± 1.6	1.81 ± 0.6	37.5 ± 5.4	44.3 ± 10	26.9 ± 14	−31.1 ± 6.9	−26.9 ± 12

a percentage change reported as a percent of control scaffolds without cell culture

## Data Availability

Data for this article, including data for cell proliferation, mechanics and protein expression are available at Mendeley Data at https://doi.org/10.17632/ynwydgkdz2.1
