# Supplementary material for: Distinct Roles of Nanoparticle Incorporation and Polymer Degradation in Fibroblast Response to Device Design
Source: Biomed Mater Devices. Author manuscript; Available in PMC 2026 Nov 5. (PMC13734723; doi:10.1007/s44174-026-00689-y)
Supplement: Supplementary file 1 [file NIHMS2215574-supplement-Supplemental_info.docx]

**Supplemental Information** for

**Distinct Roles of Nanoparticle Incorporation and Polymer Degradation in Fibroblast Response to Device Design**

in Biomedical Materials and Devices

*Kendell M Pawelec^1,2^, Erik M Shapiro^1,2,3,4,5^*

^1^ Michigan State University, Dept Radiology, East Lansing, MI 48823

^2^ Michigan State University, Institute for Quantitative Health Science and Engineering (IQ), East Lansing, MI 48823

^3^ Michigan State University, Dept Biomedical Engineering, East Lansing, MI 48823

^4^ Michigan State University, Dept of Chemical Engineering and Materials Science, East Lansing, MI 48823

^5^ Michigan State University, Dept of Physiology, East Lansing, MI 48823

S1. TaO_x_ Nanoparticle Characterization

Hydrophobic TaO_x_ nanoparticles were manufactured based on our previous procedure with one minor change.^1^ Hydrophobicity was imparted by coating nanoparticles with hexadecyltriethoxysilane (HDTES, Gelest Inc, cat no SIH5922.0). The TaO_x_ nanoparticles were imaged via transmission electron microscopy, **Figure S1**. They were spherical particles, with a mean diameter of 6.5 ± 3nm when analyzed via Image J.

**Figure S1**. Hydrophobic TaO_x_ nanoparticle characterization. (a) Visualized via transmission electron microscopy, the particles show a tight distribution in size (3-9nm) at all magnifications. (b) The amount of nanoparticles incorporated within devices, measured via TGA, was between 10-15% of the nominal value.

Thermal gravimetric analysis (TGA) was used to characterize the wt% of TaO_x_ nanoparticles in dichloromethane (DCM) after manufacture and the final wt% of TaO_x_ incorporated into polymeric devices. To determine initial wt% TaO_x_, the DCM solution was massed in an alumina pan and allowed to dry before placing in a TA Q500 (TA Instruments). For wt% TaO_x_ in devices, 12-14 mg of dry material was massed in an alumina pan. Samples were then heated to 600°C at a rate of 10°C/min under a nitrogen environment. The percentage of nanoparticles was calculated as the difference between the initial and final mass; for solutions the initial mass was the mass of the DCM solution added to the pan.

S2. Fibroblast Distribution within Devices

Devices cultured with fibroblasts for two and four weeks were fixed in 3.7% paraformaldehyde for 15 minutes. The devices were then incubated in phosphate buffered saline with 0.1% Tween-20 (PBST) for 30 minutes and permeabilized in 0.1wt% TritonX-100 for 10 minutes, while rotating. After 2 PBST washes, 5 minutes each, devices were blocked in 5wt% bovine serum albumin (BSA)/PBS for 2 hours. After 2 washes in phosphate buffered saline (PBS), the devices were incubated in HCS Nuclear Mask Red (1:1000 dilution, cat no 2738406) for 3 hours to stain cell nuclei. Devices were washed in PBS and frozen on dry ice in Tissue-Tek Optimal Cutting Temperature (OCT) Compound. Once frozen, the blocks were placed on an Emit Xerra. Images were taken of the block every 20µm. The slices were reconstructed into a three-dimensional view of the block with embedded devices. Representative cross-sections of individual devices were isolated using Image J, Figure S2.

Figure S2. Human primary dermal fibroblasts were cultured on porous devices for up to four weeks and showed a typical pattern of proliferation, with the greatest number of cells at the top and sides of the devices. Cell nuclei were stained blue prior to sectioning. In all panels, the top image is a cross-section through the device thickness, with one or two slices parallel to the device top surface. All devices were originally the same size: 4.7mm diameter, 1.5mm thick.

S3. Protein Expression

Western blotting was used to quantify protein expression. Co-cultures were lysed in Ripa buffer (supplemented with phosphatase inhibitors and protease inhibitors), and spun down at 15,000×g for 5 min at 4 °C. After which, supernatants were collected and the protein concentration of the supernatant was measured (BioRad, BCA kit). Supernatants were combined with 2× Laemmli buffer, incubated at 60°F for 20 min, separated by SDS/ PAGE (4 μg of protein per lane), and transferred to PVDF membranes. Transfer was accomplished using the iBlot 2 Gel Transfer Device (Invitrogen, IB21001), a dry transfer system, at the recommended setting of 20 V for 1 min, 23 V for 4 min and 25 V for 2 minutes (7 min total). PVDF membranes were blocked with 5% dried milk in PBST (phosphate buffered saline pH 7.4, containing 0.1% Tween-20) and probed with primary antibodies specific for alpha smooth muscle actin (αSMA, 1:1,000, Abcam, ab7817), collagen Type I (1:1000; Life Technologies, PA595137), integrin α_V_ (1:1000, Abcam, ab302640), integrin β_5_ (1:500, Invitrogen, PA5-50991), integrin β1 (1:1500, Abcam, ab179471), and vinculin (1:10000, Abcam, ab129002) at 4 °C overnight. The next day, the membrane was washed and incubated with anti-chicken IgY-HRP (12-341, Millipore) or anti-rabbit IgG-HRP (ab205718, abcam) at room temperature for 2 hr and further washed in PBST six times.

Protein bands were visualized with ECL Plus substrate (cytiva, RPN2232) on a Li-Cor Odyssey FC and resulting signal was quantified via Image J. Membranes were then washed in PBS and stripped for 15 minutes in Restore Western Blot Stripping Buffer (Thermo Scientific 21059) with subsequent PBS washing. Membranes were either blocked and reprobed or were stained for total protein using the BLOT-Fast Stain (G Biosciences, cat# 786-34) according to manufacturer’s directions. The total protein signal was imaged under visible light on a c300 imager (azure biosystems).

Signal for total protein in each lane was quantified on Image J, and the protein loading was normalized to the signal from the first lane. The normalized signal was used to adjust the measured band intensity from antibody staining, also quantified using Image J. For comparison of nanoparticle effect and matrix protein, all protein expression was recorded as a fold change from PCL matrices, with no TGFβ supplementation, with 0wt% TaO_x ,_ at week 2. The final protein quantification reported is the result of three biological replicates (2 technical replicates each). Samples of PCL + 0wt% TaO_x_ at week 2 were present on all blots and used to normalize differences between membrane exposure.

In the following figures (**Figure S3-S13**), each set of membranes is presented with all protein bands shown, along with the total protein staining for each membrane. For integrin and collagen Type I expression, only bands at high molecular weight, corresponding to the mature protein, were quantified. In cases where the protein ladder was disproportionately dark compared to the band signal, the molecular weight of bands was determined from an initial blot, with a molecular weight marker present, and the ladder was omitted in subsequent blots.

Protein expression across all scaffold groups and timepoints is presented in **Figure S14**. In the graphs, a dashed line is used to indicate no difference in expression (fold change of 1 compared to PCL + 0wt% TaO_x_ at week 2). Fold changes below 1 represented decrease in protein expression, while fold changes above 1 were seen as increases in expression.

**Figure S3:** Western blots of porous devices at week 2 (biological replicate 1). Top row: total protein, second and third rows (A) integrin β_1_, (B) αSMA, vinculin, (C) integrin α_V_, (D) collagen Type I. The sample loaded in each lane is specified at the bottom. *Indicates a band remaining from previous staining, before stripping and reprobing the membranes.

**Figure S4:** Western blots of porous devices at week 2 (biological replicate 2). Top row: total protein, second and third rows (A) integrin β_1_, (B) αSMA, vinculin, (C) integrin α_V_, (D) collagen Type I. The sample loaded in each lane is specified at the bottom.

**Figure S5:** Western blots of porous devices at week 2 (biological replicate 3). Top row: total protein, second and third rows (A) integrin β_1_, (B) αSMA, vinculin, (C) integrin α_V_, (D) collagen Type I. The sample loaded in each lane is specified at the bottom.

**Figure S6:** Western blots of porous devices at week 4 (biological replicate 1). Top row: total protein, second and third rows (A) integrin β_1_, (B) αSMA, vinculin, (C) integrin α_V_, (D) collagen Type I. The sample loaded in each lane is specified at the bottom.

**Figure S7:** Western blots of porous devices at week 4 (biological replicate 2). Top row: total protein, second and third rows (A) integrin β_1_, (B) αSMA, vinculin, (C) integrin α_V_, (D) collagen Type I. The sample loaded in each lane is specified at the bottom.

**Figure S8:** Western blots of porous devices at week 4 (biological replicate 3). Top row: total protein, second and third rows (A) integrin β_1_, (B) αSMA, vinculin, (C) integrin α_V_, (D) collagen Type I. The sample loaded in each lane is specified at the bottom.

**Figure S9:** Western blots of porous devices at week 2: (A) biological replicate 1 (B) biological replicate 2 (C) biological replicate 3. Top row: total protein, second row: integrin β_5._ The sample loaded in each lane is specified in the key. Protein signal was low compared to the protein ladder, so it was excluded in all blots except C.

**Figure S10:**  Western blots of porous devices at week 4: (A) biological replicate 1 (B) biological replicate 2 (C) biological replicate 3. Top row: total protein, second row: integrin β_5_The sample loaded in each lane is specified in the key. Protein signal was low compared to the protein ladder, so it was excluded in all blots except C.

**Figure S11:** Western blots of films with lactic acid supplementation at week 2 (biological replicate 1). Top row: total protein, second row: (A) collagen Type I, integrin α_V_, (B) αSMA, integrin β_1_. The sample loaded in each lane is specified in the key.

**Figure S12:** Western blots of films with lactic acid supplementation at week 2 (biological replicate 2). Top row: total protein, second row: (A) collagen Type I, integrin α_V_, (B) αSMA, integrin β_1_. The sample loaded in each lane is specified in the key.

**Figure S13:** Western blots of films with lactic acid supplementation at week 2 (biological replicate 3). Top row: total protein, second row: (A) collagen Type I, integrin α_V_, (B) αSMA, integrin β_1_. The sample loaded in each lane is specified in the key.

**Figure S14:** Protein expression across all tested scaffold groups at weeks 2 and 4: (a) αSMA, (b) vinculin, (c) integrin α_V_, (d) integrin β_1_, (e) integrin β_5_, (f) collagen Type I. α < 0.05, * p < 0.05, ** p < 0.01, *** p < 0.001, $ significantly different from all other groups at timepoint (p<0.05), ## significantly different from all other groups at timepoint (p<0.01), ^ significantly different from all other groups at timepoint (p<0.001), # significantly different from all other groups at timepoint (p<0.0001), & significantly lower than PLGA groups at timepoint (p<0.05).

**References**

1 S. Chakravarty, J. M. L. Hix, K. A. Wiewiora, M. C. Volk, E. Kenyon, D. D. Shuboni-Mulligan, B. Blanco-Fernandez, M. Kiupel, J. Thomas, L. F. Sempere, and E. M. Shapiro. *Nanoscale*, 2020, **12**, 7720–7734.
